# MEF2C Alleviates Postoperative Cognitive Dysfunction by Repressing Ferroptosis

**DOI:** 10.1111/cns.70066

**Published:** 2024-09-30

**Authors:** Shanshan Wang, Zankai Wu, Xueshan Bu, Xuan Peng, Qin Zhou, Wenqin Song, Wenwei Gao, Wei Wang, Zhongyuan Xia

**Affiliations:** ^1^ Department of Anesthesiology Renmin Hospital of Wuhan University Wuhan Hubei China; ^2^ Department of Breast and Thyroid Surgery Renmin Hospital of Wuhan University Wuhan Hubei China; ^3^ Department of Critical Care Medicine Renmin Hospital of Wuhan University Wuhan Hubei China

**Keywords:** ferroptosis, glutathione peroxidase 4, myocyte‐specific enhancer factor 2C, postoperative cognitive dysfunction

## Abstract

**Background:**

Ferroptosis, a form of programmed cell death featured by lipid peroxidation, has been proposed as a potential etiology for postoperative cognitive dysfunction (POCD). Myocyte‐specific enhancer factor 2C (MEF2C), a transcription factor expressed in various brain cell types, has been implicated in cognitive disorders. This study sought to ascertain whether MEF2C governs postoperative cognitive capacity by affecting ferroptosis.

**Methods:**

Transcriptomic analysis of public data was used to identify MEF2C as a candidate differentially expressed gene in the hippocampus of POCD mice. The POCD mouse model was established via aseptic laparotomy under isoflurane anesthesia after treatment with recombinant adeno‐associated virus 9 (AAV9)‐mediated overexpression of MEF2C and/or the glutathione peroxidase 4 (GPX4) inhibitor RSL3. Cognitive performance, Nissl staining, and ferroptosis‐related parameters were assessed. Dual‐luciferase reporter gene assays and chromatin immunoprecipitation assays were implemented to elucidate the mechanism by which MEF2C transcriptionally activates GPX4.

**Results:**

MEF2C mRNA and protein levels decreased in the mouse hippocampus following anesthesia and surgery. MEF2C overexpression ameliorated postoperative memory decline, hindered lipid peroxidation and iron accumulation, and enhanced antioxidant capacity, which were reversed by RSL3. Additionally, MEF2C was found to directly bind to the *Gpx4* promoter and activate its transcription.

**Conclusions:**

Our findings suggest that MEF2C may be a promising therapeutic target for POCD through its negative modulation of ferroptosis.

## Graphical abstract

MEF2C overexpression alleviates postoperative cognitive dysfunction by transcriptionally activating *Gpx4* and suppressing ferroptosis.

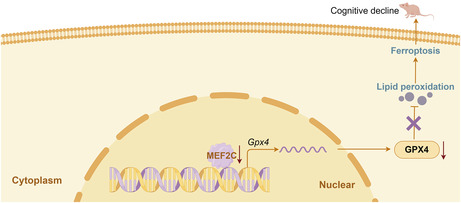

**Abbreviations**
AAV9: adeno‐associated virus 9
AD: Alzheimer's disease
BCA: bicinchoninic acid
BP: biological process
CA1: cornu ammonis 1
CaMKIIα: calcium/calmodulin‐dependent kinase IIα
ChIP: chromatin immunoprecipitation
DAZAP1: deleted in zoospermia‐associated protein 1
DEG: differentially expressed gene
DMSO: diluted in dimethyl sulfoxide
ECL: enhanced chemiluminescence
Gata3: GATA binding protein 3
GEPIA: Gene Expression Profiling Interactive Analysis
GO: Gene Ontology
GPX4: glutathione peroxidase 4
GRIN1: glutamate lonotropic receptor NMDA type subunit 1
GSH: reduced glutathione
HSPB1: heat shock protein beta‐1
KEGG: Kyoto Encyclopedia of Genes and Genomes
MADS‐BOX: minichromosome maintenance 1, agamous, deficiens and serum response factor box
MDA: malondialdehyde
MEF2C: myocyte‐specific enhancer 2C
MWM: Morris water maze
NF2: neurofibromin 2
NFE2L2: nuclear factor erythroid‐2‐related factor 2
NOR: novel object recognition
PDVF: polyvinylidene difluoride
POCD: postoperative cognitive dysfunction
Rac1: Rac family small GTPase 1
Rap1: Ras‐proximate‐1
RB1: retinoblastoma 1
RIPA: radioimmunoprecipitation assay
RSL3: RAS‐selective lethal small molecule 3
RT‐qPCR: real‐time quantitative polymerase chain reaction
SDS‐PAGE: sodium dodecyl sulfate–polyacrylamide gel electrophoresis
SEM: standard error of the mean
SOD: superoxide dismutase
SYN1: synapsin 1
SYN2: synapsin 2
TF: transcription factor
TRRUST: Transcriptional Regulatory Relationships Unraveled by Sentence‐based Text mining
WB: western blot

## 1 Introduction

Postoperative cognitive dysfunction (POCD), characterized by a spectrum of impairments in information processing, social skills, mental faculties, and cognitive abilities, is an acute or persistent neurological complication in geriatric patients after major surgery [1]. The morbid state is intimately linked to increased hospitalization costs, compromised quality of life, and heightened risks of mortality and dementia [2]. Despite substantial research efforts in recent years, the pathophysiological mechanisms underlying POCD remain incompletely elucidated.

Ferroptosis is an iron‐catalyzed, non‐apoptotic pattern of cell death culminating in increased mitochondrial bilayer membrane density, cristae reduction or vanishing, and outer membrane rupture [3]. Iron deposition and lipid peroxidation are principal hallmarks of ferroptosis and cells exploit various enzymatic and nonenzymatic oxidation systems to surveil and safeguard against deleterious cytomembrane lipid peroxidation [4]. Glutathione peroxidase 4 (GPX4), a core enzyme capable of detoxifying lipid hydroperoxides in phospholipids (PL‐PUFA‐OOH) into harmless lipid alcohols using the reductant glutathione, serves as a protective factor against lipoperoxidation injury and ferroptosis [5]. Mounting evidence implies that ferroptosis is responsible for a battery of neurological disorders, including cerebral ischemia/reperfusion injury [6, 7], Alzheimer's disease (AD) [8], and POCD [9]. Several compounds or medications have been documented to attenuate postoperative cognitive deficits by dampening ferroptosis [10, 11, 12].

The transcription factor (TF) myocyte enhancer factor 2C (MEF2C) encompasses the N‐terminal minichromosome maintenance 1, agamous, deficiens and serum response factor box (MADS‐BOX), and MEF2 domain, which mediate DNA binding and protein dimerization [13]. It is abundantly expressed in the cerebellum, cerebral cortex, and hippocampus of both humans and mice [14]. MEF2C has been implicated in neuronal differentiation, axon pruning, dendritic remodeling, and synaptic plasticity [15]. Excessive microglial immune responses were observed in the hippocampus of *Mef2c*‐deficient mice following exposure to interferon‐β and pentobarbital/laparotomy [16, 17]. Neuronal MEF2C ameliorated cognitive deterioration associated with AD progression by transcriptionally activating genes related to synaptic plasticity, such as CaMKIIα, SYN1, SYN2, and GRIN1 [18, 19]. MEF2C has been elaborated to inhibit neuronal apoptosis and provide protection against POCD through its interaction with the promoter of miR‐106a [20]. Recent studies have shown that MEF2C confers resistance to ferroptosis in meningioma by upregulating the transcription of NF2 and E‐Cadherin [21]. However, it remains unknown whether MEF2C exerts favorable effects on postoperative cognitive capacity via transcriptionally altering ferroptosis suppressor genes.

The objective of our research was to elucidate the potential involvement of ferroptosis in the neurocognitive benefits of MEF2C. Furthermore, we investigated the regulatory interactions between MEF2C and ferroptosis suppressor genes in the development of POCD.

## 2 Materials and Methods

### 2.1 Identification of the Differentially Expressed Genes and Functional Enrichment Analysis

The whole‐transcriptome sequencing data retrieved from Zhang et al. [22] included six pairs of hippocampi from control and anesthesia/surgery‐treated mice. Differentially expressed genes (DEGs) were identified using a cutoff standard of *p* < 0.05 and |log_2_FC| > 3. Enrichment analysis of the DEGs was implemented utilizing the web‐based platform Metascape (http://metascape.org), a bioinformatics pipeline specifically designed for comprehensive gene function annotation based on Gene Ontology (GO) biological processes (BP) and Kyoto Encyclopedia of Genes and Genomes (KEGG) pathways [23]. To understand the function of TFs in POCD, overlapping mRNAs between the DEGs and mouse TFs derived from the online TRRUST database that consolidates numerous public TFs [24] were considered DETFs. The Venn diagram was utilized to overlap the DETFs and DEGs involved in at least 50 BP terms.

### 2.2 Antibodies and Reagents

The antibodies utilized in the Western blot (WB) analysis included MEF2C (#ab211493, 1:1000; Abcam), GPX4 (#67763‐1‐Ig, 1:2000; Proteintech), and glyceraldehyde‐3‐phosphate dehydrogenase (GAPDH; #ab181602, 1:10,000; Abcam). Horseradish peroxidase (HRP)‐conjugated secondary antibodies (1:10,000) were sourced from ASPEN (Wuhan, China). Isoflurane was procured from Abbott Laboratories (Shanghai, China). The GPX4 inhibitor RSL3 (#S8155; Selleck, Houston, TX, USA) was initially dissolved in dimethyl sulfoxide (DMSO) to a concentration of 20 mg/mL and subsequently diluted with corn oil to achieve a final concentration of 1 mg/mL. Commercial kits for malondialdehyde (MDA), reduced glutathione (GSH), superoxide dismutase (SOD), and iron were purchased from Nanjing Jiancheng Bioengineering Institute (Nanjing, China).

### 2.3 Animals

The animal study protocols received approval from Renmin Hospital of Wuhan University, adhering to the Guide for the Care and Use of Laboratory Animals (No.: WDRM‐20230701E). Specific pathogen‐free male C57BL/6 mice, aged 12 months and weighing 28–32 g, were procured from the Hubei Provincial Centers for Disease Control and Prevention. Animals were acclimatized for 7 days in a controlled environment (consistent temperature and humidity, and a 12‐h light/dark cycle) with ad libitum access to chow and water.

### 2.4 Adeno‐Associated Virus 9 Transduction

The recombinant adeno‐associated virus 9 (AAV9) vector encoding *Mef2c* (AAV9‐*Mef2c*) was supplied by OBiO Biotechnology Co. Ltd. (Shanghai, China). Anesthetized animals were secured in a stereotaxic frame, and a volume of 2 μL of AAV9‐*Mef2c* was microinjected into the bilateral cornu ammonis (CA) 1 region of the hippocampus at a rate of 0.1 μL/min. The injection sites were as follows: anterioposterior (AP) −2.0 mm, mediolateral (ML) ±2.3 mm, dorsoventral (DV) −2.0 mm [1, 25]. The animals were allowed to recover from the viral transduction for 2 weeks before POCD modeling.

### 2.5 Animal Model

Based on our previous studies [1, 26, 27], laparotomy under isoflurane anesthesia was conducted to imitate POCD. Briefly, animals were anesthetized using a Matrx anesthesia machine (Midmark, Dayton, OH, USA) prefilled with 1.5%–3% isoflurane and 100% oxygen. Laparotomy was executed via a 1.5‐cm longitudinal abdominal incision through the skin, muscles, and peritoneum. Approximately 5 cm of the small intestine was exposed and gently manipulated for 10 min using saline‐soaked sterile gauze. Finally, the wound was sutured using absorbable sutures in a layered fashion. The entire surgical procedure lasted about 30 min. An electric blanket was used to avoid intraoperative hypothermia. Compound lidocaine cream provided appropriate postoperative analgesia until behavioral experiments were performed.

### 2.6 Experimental Groups

The experimental design was divided into two distinct parts. For Experimental Part I, animals were randomly assigned to one of three groups: Control (C) group: mice inhaled pure oxygen without undergoing anesthesia or surgery; Anesthesia and Surgery (AS) group: mice were subjected to laparotomy under isoflurane anesthesia; and AS + MEF2C group: mice received AAV9‐*Mef2c* 14 days before abdominal surgery (Figure 2A). Experimental Part II comprised five groups, as depicted in Figure 5A: C group; AS group; AS + MEF2C group; AS + RSL3 group: mice were intraperitoneally administered RSL3 (5 mg/kg) [28] once daily for 3 days before POCD modeling; AS + MEF2C + RSL3 group: mice were administered AAV9‐*Mef2c* 2 weeks prior to undergoing abdominal surgery and received RSL3 once daily for 3 days before modeling.

### 2.7 Prediction for the Target Genes of MEF2C

Harmonizome (https://maayanlab.cloud/Harmonizome/), a comprehensive portal of knowledge about genes and proteins [29], was utilized to predict target genes regulated by MEF2C. As a transcriptional activator, MEF2C has been shown to inhibit ferroptosis in tumor cells by upregulating the ferroptosis suppressor gene NF2 [21]. FerrDb (http://www.zhounan.org/ferrdb/legacy/), an integrated resource for ferroptosis regulators and ferroptosis‐disease associations [30], was employed to obtain the ferroptosis suppressor genes. The intersection between the ferroptosis suppressor genes and target genes was visualized using a Venn diagram. GEPIA, a web server for normal gene expression profiling and interactive analysis [31], was used to analyze the correlation between MEF2C and candidate target genes.

### 2.8 Novel Object Recognition Test

The animals underwent a 30‐min acclimation period to the experimental environment on the fourth‐day post‐surgery. During the familiarization phase, each mouse was allowed for 5 min to freely explore two identical cuboids at diagonally opposite corners within a 50 × 50 × 40 cm^3^ open field apparatus. Twenty‐four hours later, one of the cuboids was substituted by a new cylinder (5‐cm diameter and 15‐cm height). The discrimination index was defined as the cumulative time spent exploring the novel object divided by the total time spent exploring both the novel and familiar objects [32].

### 2.9 Morris Water Maze Test

The Morris water maze (MWM) test was conducted 7–12 days after surgery to investigate the hippocampus‐dependent spatial memory as previously described [33]. The cylindrical pool, measuring 120 cm in diameter and 50 cm in height, was equipped with a 6‐cm‐diameter concealed platform in the fourth quadrant. The MWM test was composed of two phases: a location navigation phase, conducted four times daily for five consecutive days, and a space exploration phase without the platform. Mice were randomly introduced into the water from one of the four quadrants to find the platform within 60 s; if unsuccessful, they were guided to the platform manually. The duration required to locate the platform was measured as the escape latency. The probe trial was performed 12 days post‐surgery when the platform was removed. The swimming velocity, time spent in the fourth (target) quadrant, and platform crossing times were automatically documented using a video tracking equipment (XinRuan, Shanghai, China).

### 2.10 Nissl Staining

Following behavioral tests, three mice were randomly selected from each group and euthanized under deep anesthesia using isoflurane to isolate hippocampal CA1 tissues on ice. Paraffin sections were then dehydrated using a series of alcohol gradients, followed by incubation with Nissl staining solution for 10 min. The sections were cover‐slipped with neutral resin and observed under a light microscope (Olympus, Tokyo, Japan) by an investigator blinded to the intervention. The number of surviving neurons in the bilateral hippocampi of each mouse was quantified using ImageJ software.

### 2.11 Western Blot Analysis

Proteins were extracted from hippocampal CA1 tissues using radioimmunoprecipitation assay (RIPA) buffer (ASPEN, Wuhan, China). The lysates were separated by sodium dodecyl sulfate–polyacrylamide gel electrophoresis (SDS‐PAGE; ASPEN), followed by electrotransfer onto polyvinylidene difluoride (PVDF) membranes (Millipore, USA). The specimens were then incubated with appropriate primary antibodies and corresponding secondary antibodies. Finally, immunodetection was implemented using an enhanced chemiluminescence (ECL) assay kit (ASPEN).

### 2.12 Real‐Time Quantitative Polymerase Chain Reaction

Total RNA was extracted from hippocampal CA1 tissues using the TRIpure reagent (ELK, Wuhan, China), followed by cDNA synthesis using the EntiLink 1st Strand cDNA Synthesis Super Mix (ELK). PCR amplification was conducted on a QuantStudio 6 Flex System (Life Technologies, Carlsbad, CA, USA). The primer sequences for *Mef2c* and *Gpx4* were as follows: 5′‐GCCAGCTGGTCTGGCTTTAT‐3′ (sense) and 5′‐GCTTCACTTCATCTCTCCAGAGG‐3′ (antisense), and 5′‐CAGGAGCCAGGAAGTAATCAAG‐3′ (sense) and 5′‐AAGTTCCATTTGATGGCATTTC‐3′ (antisense), respectively. For *Gapdh*, the primer sequences were 5′‐TGAAGGGTGGAGCCAAAAG‐3′ (sense) and 5′‐AGTCTTCTGGGTGGCAGTGAT‐3′ (antisense). The relative mRNA expression of *Gpx4* was normalized to that of *Gapdh* using the $2^{-\Delta \Delta C_{t}}$ method.

### 2.13 Measurement of MDA, GSH, SOD, and Iron Levels

Hippocampal homogenates were prepared for protein concentration determination using a bicinchoninic acid (BCA) assay kit (ASPEN) and subsequently centrifuged for 10 min. The resulting supernatant was harvested for the quantification of MDA, GSH, SOD, and iron levels according to the manufacturer's instructions for the respective assay kits. The optical density readings for MDA, GSH, SOD, and iron were measured at wavelengths of 532, 405, 450, and 520 nm, respectively, using a microplate reader (Diatek, Wuxi, China).

### 2.14 Dual‐Luciferase Report Gene Assay

The GPX4 promoter sequence was analyzed using the PROMO database [34] (https://alggen.lsi.upc.es/cgi‐bin/promo_v3/promo/promoinit.cgi?dirDB=TF_8.3) to predict its binding sites with MEF2C. The wild‐type *Gpx4* promoter sequence was amplified by PCR and inserted into the pGL3‐basic vector to construct the luciferase reporter plasmid (ELK). HT22 neurons were maintained in Dulbecco's modified Eagle's medium (DMEM; Hyclone, USA) and transfected with the pGL3‐basic‐*Gpx4* promoter, along with either the pcDNA3.1‐empty vector or pcDNA3.1‐*Mef2c*. After 48 h of transfection, the relative luciferase activity was assessed using a luciferase reporter gene assay kit (Beyotime, Shanghai, China).

### 2.15 Chromatin Immunoprecipitation Coupled With Quantitative PCR (ChIP‐qPCR) Assay

HT22 neurons were fixed in formaldehyde at room temperature to crosslink DNA and TFs. The cells were scraped into an SDS lysis buffer and sonicated to obtain chromatin fragments. Immunoprecipitation was performed by incubating HT22 cells with either an anti‐MEF2C antibody (1:50) or a nonspecific IgG. Subsequently, the purified DNA sequences were identified using real‐time quantitative polymerase chain reaction (RT‐qPCR) with specific primers designed against the *Gpx4* promoter region. The primer sequences used for the *Gpx4* promoter region were 5′‐GATCTCTGAGGCCAGCCTG‐3′ (sense) and 5′‐TACTGGCTCCCAGGAAGCTAG‐3′ (antisense).

### 2.16 Statistical Analyses

The Shapiro–Wilk test was employed to assess the normality of the distribution of results. Values were presented as mean ± standard error of the mean (SEM) and analyzed using an independent *t*‐test, one‐way analysis of variance (ANOVA) followed by Tukey's post hoc test, or two‐way ANOVA with Bonferroni's multiple comparisons test in GraphPad Prism version 9.0 (GraphPad Software Inc., San Diego, CA, USA). Statistical significance was defined as *p* < 0.05.

## 3 Results

### 3.1 MEF2C Was Downregulated in the Hippocampus of Mice Undergoing Anesthesia and Surgery

**FIGURE 1:**
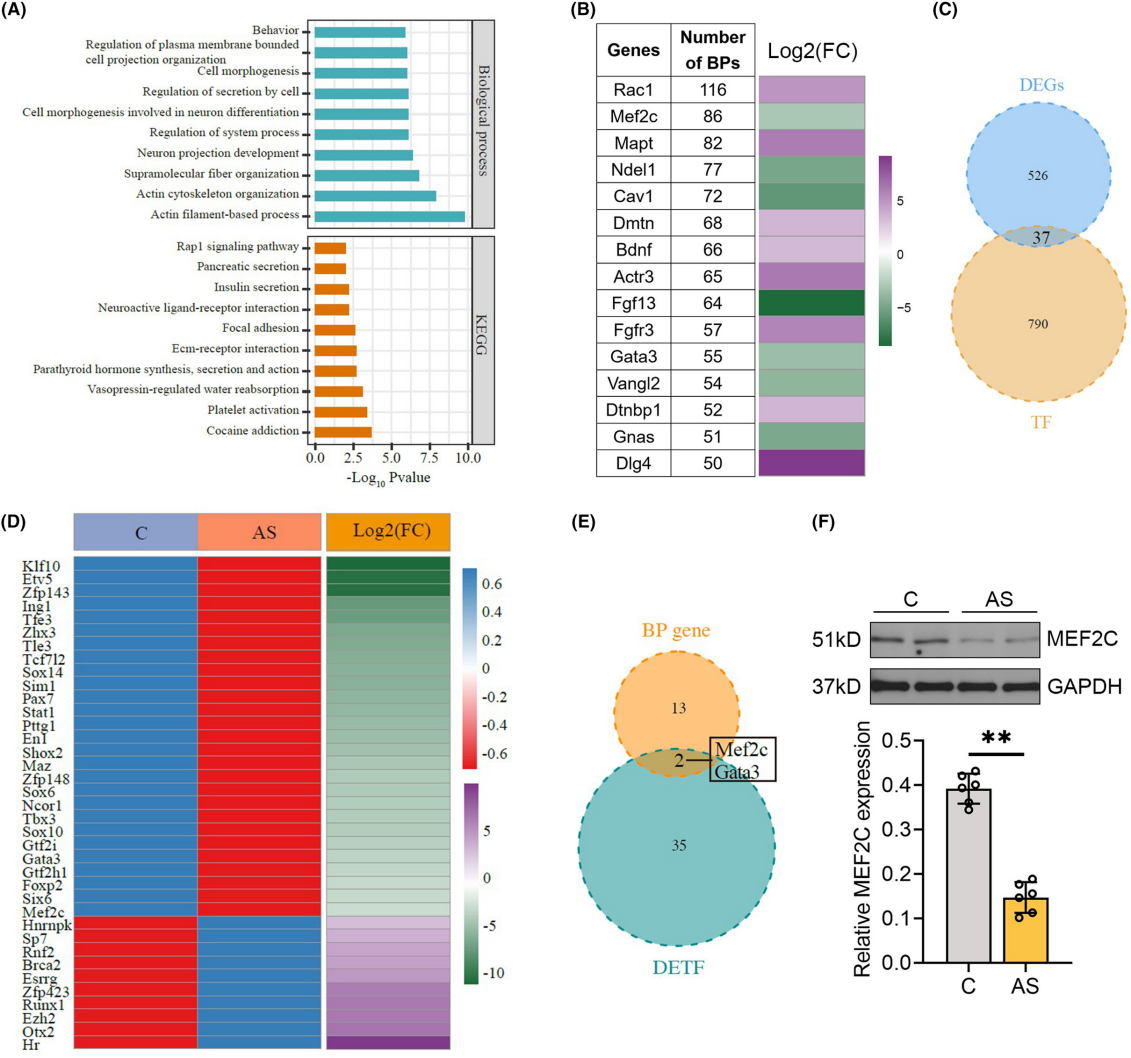
Anesthesia and surgery diminished the expression of MEF2C in the hippocampus of mice. (A) Functional annotation and KEGG pathway enrichment analysis of the DEGs between control mice and anesthesia/surgery‐treated mice. (B) Fifteen DEGs involved in more than 50 biological processes (left table) and the log_2_ fold‐change values of genes (right panel). (C) Venn diagram of 37 intersected transcription factors with DEGs. (D) Heatmap plots of the 37 differentially expressed transcription factors (DETFs). (E) Venn diagram of intersected DETFs with the 15 genes enriched in over 50 biological processes. (F) Western blot analysis of MEF2C protein expression in the hippocampus of mice following anesthesia and surgery (AS) treatment. Densitometric quantification normalized to GAPDH was denoted as mean ± SEM (*n* = 6). **p < 0.01, unpaired *t*‐test.

To gain insight into the pathogenesis of POCD, we analyzed the online hippocampus RNA‐seq data from six pairs of control mice and anesthesia/surgery‐treated mice [22]. A total of 563 DEGs with thresholds of |log_2_FC| > 3 and *p* value < 0.05 were identified in the hippocampus of AS mice, of which 219 were upregulated and 344 were downregulated. The enriched GO biological process terms primarily pertained to neuron differentiation and neuron projection development. Moreover, KEGG pathway enrichment analysis revealed that the DEGs were predominantly enriched in the Rap1 signaling, neuroactive ligand‐receptor interaction, platelet activation, and cocaine addiction (Figure 1A). Fifteen genes enriched in at least 50 BP terms were ranked from most to least, of which eight were upregulated and seven were downregulated. Specifically, *Rac1* was involved in 116 biological processes, whereas *Mef2c* participated in 86 biological processes (Figure 1B). Given the pivotal role of transcriptional regulation in orchestrating and modulating various neural functions, such as neuronal migration, axonal outgrowth, dendritic branching, and synaptogenesis [35], we further screened 37 overlapping TFs by integrating TRRUST database and DEGs, of which 10 were upregulated and 27 were downregulated (Figure 1C,D). There were two TFs (*Mef2c* and *Gata3*) among 15 DEGs involved most in biological processes (Figure 1E). According to single‐cell brain transcriptome data (GSE67835 and Brain RNA‐Seq) [36, 37], *Gata3* was excluded because of its low abundance in neurons and neuroglia (Figure S1A–C). Consequently, *Mef2c* was finally identified as the candidate TF. Isoflurane anesthesia and laparotomy significantly decreased MEF2C mRNA and protein expression in the hippocampus of mice (Figure S1D, Figure 1F).

### 3.2 MEF2C Overexpression Alleviated Cognitive Impairment Induced by Isoflurane and Laparotomy

**FIGURE 2:**
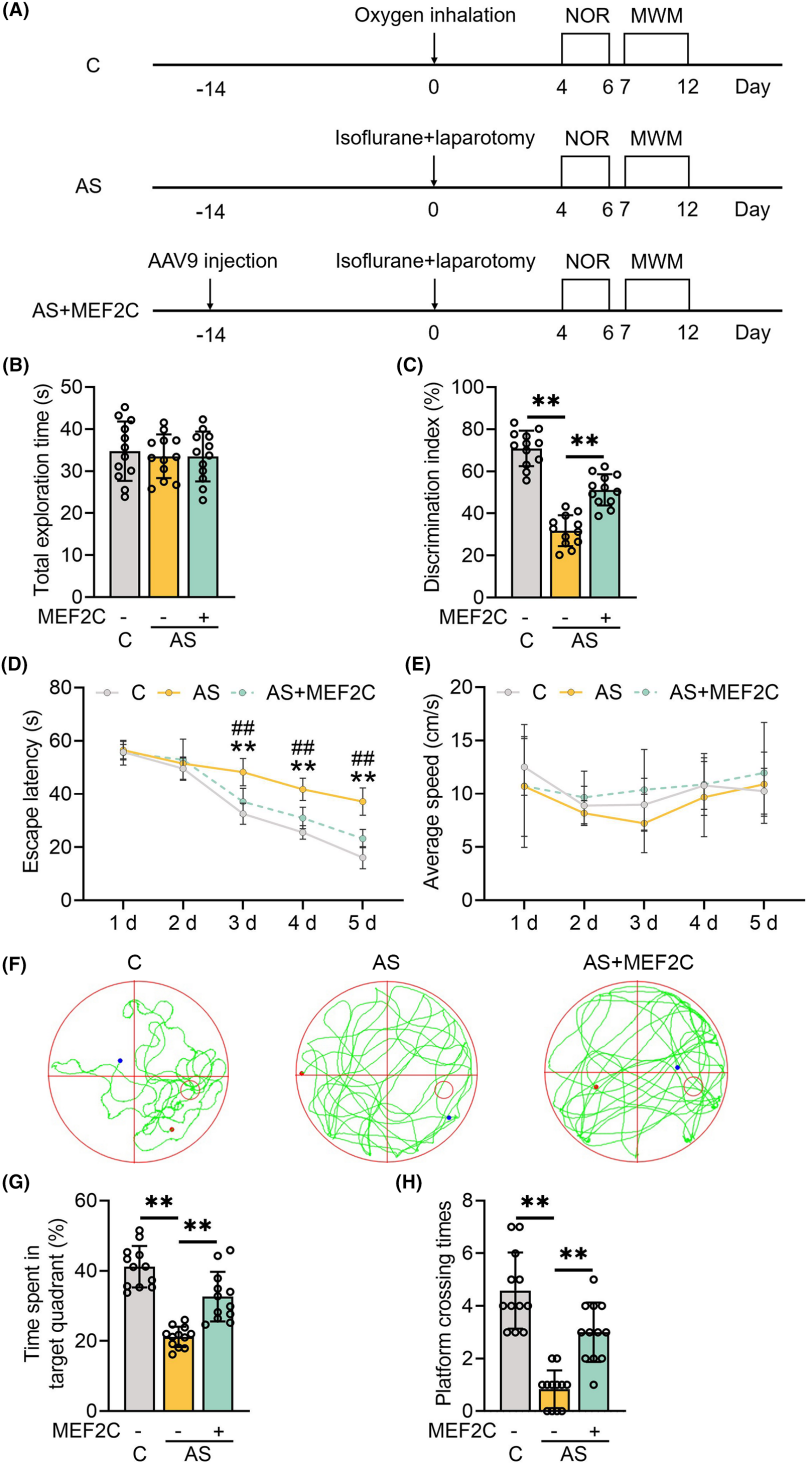
MEF2C overexpression mitigated postoperative cognitive disorders. (A) Schematic diagram of the experimental design. Total exploration time (B) and discrimination index (C) in the NOR test. Escape latency (D) and average swimming speed (E) during the acquisition phase. Two‐way ANOVA followed by Bonferroni's multiple comparisons test. **p < 0.01, C versus AS; ^##^ *p* < 0.01, AS versus AS + MEF2C. (F) The trajectory of mice following the indicated treatments. The red circle denotes the hidden platform, the red and blue dots denote the start and end of swimming, respectively. Time spent in the target quadrant (G) and platform crossing times (H) during the space exploration test of the MWM test. One‐way ANOVA followed by Tukey's post hoc test. **p < 0.01. Data were denoted as mean ± SEM (*n* = 12).

To substantiate the role of MEF2C in the occurrence and development of POCD, recombinant AAV9 encoding *Mef2c* was delivered in mice to yield wide‐scale expression in the hippocampus. The novel object recognition (NOR) test showed no significant difference in the total exploration time among the three groups (Figure 2B). The discrimination index was substantially reduced following exposure to isoflurane and laparotomy, whereas anesthesia/surgery‐treated mice receiving injection of AAV9‐*Mef2c* visibly preferred the novel object (Figure 2C). During the training sessions of the MWM test, anesthesia and surgery significantly prolonged the escape latency of animals on the 3rd, 4th, and 5th day. However, mice receiving AAV9‐*Mef2c* therapy spent shorter time reaching the hidden platform compared with the AS group (Figure 2D). There was no discernible difference in the average swimming speed among the three groups, indicating no impact of anesthesia/surgery, regardless of AAV9‐*Mef2c* pretreatment, on spontaneous locomotor activity in rodents (Figure 2E). During the probe trial, mice undergoing abdominal procedure under isoflurane anesthesia exhibited obviously worse performance compared with the control group, as evidenced by reduced target quadrant stay and platform crossing times. In contrast, MEF2C overexpression significantly increased the average target quadrant residence time and platform crossing frequency (Figure 2F–H). These results indicate that the POCD model is successfully established and MEF2C exerts neuroprotective effects against isoflurane/laparotomy‐provoked cognitive disturbances.

### 3.3 GPX4, a Ferroptosis Suppressor, Was Transcriptionally Activated by MEF2C in HT22 Neurons

**FIGURE 3:**
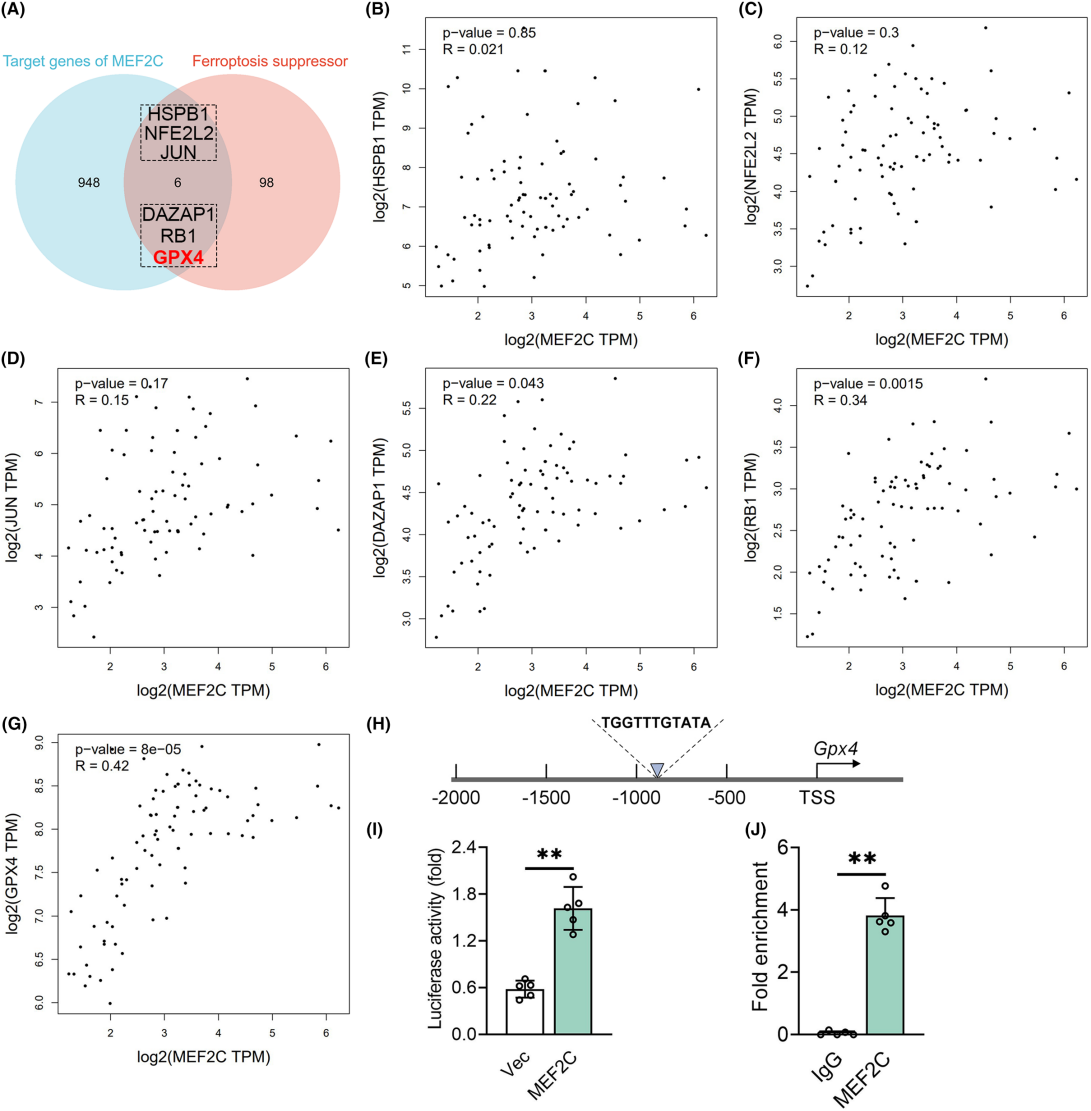
GPX4 was the target gene of MEF2C. (A) Venn diagram of six intersected ferroptosis suppressor genes with target genes predicted by the Harmonizonme database. (B–G) Correlation analysis of MEF2C with HSPB1, NFE2L2, JUN, DAZAP1, RB1, and GPX4 in the hippocampus by Gene Expression Profile Interactive Analysis (GEPIA; http://gepia.cancer‐pku.cn). (H) Schematic diagram of the putative binding sites of MEF2C in the *Gpx4* promoter predicted by the PROMO database. (I) Murine *Gpx4* promoter luciferase reporter plasmid was cotransfected into HT22 cells with the pcDNA3.1‐*Mef2c* for 48 h. The relative luciferase activity was calculated by the firefly luciferase/renilla luciferase ratio. (J) ChIP assay for the binding of MEF2C to the promoter region of the *Gpx4* gene. Data were denoted as mean ± SEM (*n* = 5). **p < 0.01, unpaired *t*‐test.

Although reduced microglial MEF2C expression contributed to POCD by augmenting the immunoreactivity of aged microglia [17], recent online single‐cell RNA‐sequencing data [36, 37] indicate that MEF2C is expressed most abundantly in neurons of both humans and mice (Figure S2A–C). MEF2C has been found to prevent neurons from apoptosis [20], but its specific target genes involved in the progression of POCD remain unclear. Emerging evidence for the anti‐ferroptotic properties of MEF2C in meningioma and lung cancer [21, 38] suggests that it may activate ferroptosis suppressor genes through transcriptional regulation. Six downstream genes of MEF2C were predicted by integrating the Harmonizome [29] and FerrDb [30] portals (Figure 3A). Furthermore, GEPIA was utilized to investigate the correlations between MEF2C and these six target genes in the hippocampus [31]. There was no statistical difference in Pearson's correlation coefficient between MEF2C and the three ferroptosis inhibitory genes HSPB1, NFE2L2, and JUN (Figure 3B–D). Despite no significant correlation between MEF2C and DAZAP1 expression levels (Figure 3E), MEF2C exhibited a positive correlation with RB1 (*R* = 0.34) and GPX4 (*R* = 0.42) (Figure 3F,G), suggesting *Gpx4* as the most probable target of MEF2C. The PROMO database was used to predict the potential binding sites between MEF2C and the promoter region of *Gpx4*. The nucleotide sequence (TGGTTTGTATA) located 890–880 bp upstream of the transcription start site (TSS) was the putative MEF2C binding site (Figure 3H). Dual‐luciferase reporter assay showed that MEF2C overexpression increased *Gpx4* promoter activity, indicating a potential interaction between MEF2C and the *Gpx4* promoter (Figure 3I). ChIP assay revealed that anti‐MEF2C, but not control IgG, specifically enriched DNA fragments containing the GPX4 promoter region (Figure 3J). These findings imply that MEF2C directly interacts with the GPX4 promoter and functions as a transcriptional activator.

### 3.4 MEF2C Overexpression Augmented GPX4 Expression and Repressed Ferroptosis

**FIGURE 4:**
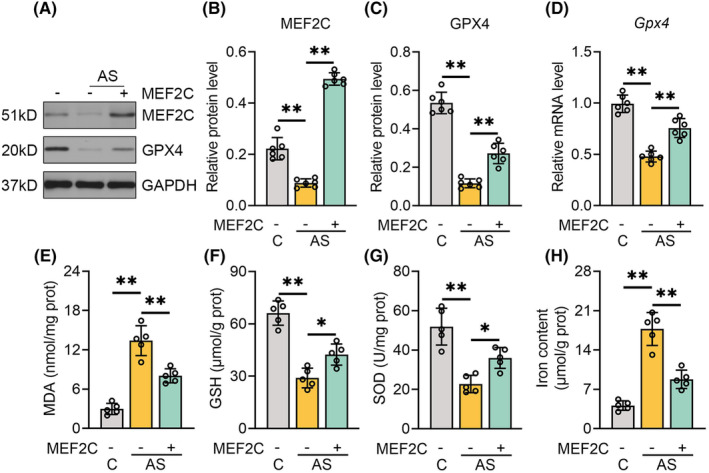
MEF2C inhibited ferroptosis induced by anesthesia and surgery. (A) Gel images of MEF2C and GPX4 in the hippocampus following the indicated treatments. (B, C) Quantification results of MEF2C and GPX4 normalized to GAPDH. (D) RT‐qPCR analysis of GPX4 mRNA expression in the hippocampus of mice following the indicated treatments. (E–H) MDA, GSH, SOD, and iron levels in the hippocampus of mice following the indicated treatments. Data were denoted as mean ± SEM (*n* = 6 or 5). *p < 0.05, **p < 0.01, one‐way ANOVA followed by Tukey's post hoc test.

To elucidate the potential involvement of ferroptosis in the neuroprotective effects of MEF2C against POCD, the levels of MDA, GSH, SOD, iron, and GPX4 were examined in hippocampal tissues. The markedly elevated MEF2C mRNA and protein expression, as confirmed by qPCR and immunoblotting, indicated successful transfection of the AAV9 vector harboring *Mef2c* (Figure S3, Figure 4A,B). Isoflurane and laparotomy prominently diminished GPX4 transcription and translation levels, which was reversed by AAV9‐*Mef2c* (Figure 4A,C,D). The lipid peroxide end‐product MDA concentration was significantly increased in the mouse hippocampi following anesthesia and surgery, which was restored by MEF2C overexpression (Figure 4E). Isoflurane/laparotomy‐treated mice showed reduced hippocampal GSH levels and antioxidant activity of SOD, whereas *Mef2c*‐OE mice exhibited stronger antioxidant capacity post‐surgery compared with the AS group (Figure 4F,G). Additionally, iron overload was observed in the hippocampus of mice exposed to isoflurane anesthesia and aseptic laparotomy, which was ameliorated by AAV9‐*Mef2c* (Figure 4H). Overall, our data underline the repressive effect of MEF2C on anesthesia/surgery‐evoked ferroptosis.

### 3.5 RSL3, a GPX4 Inhibitor, Reversed the Protective Effect of MEF2C on POCD

**FIGURE 5:**
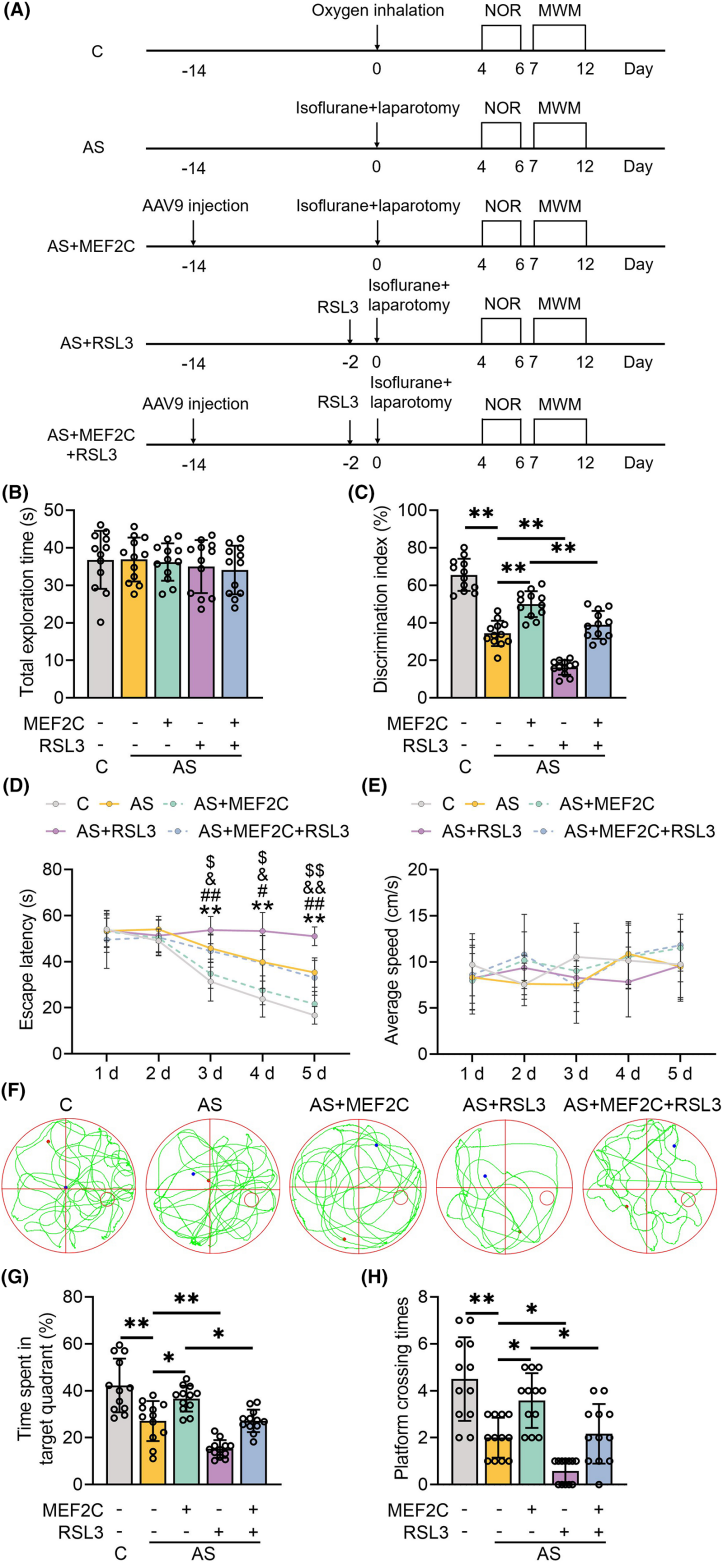
RSL3 abolished the favorable effect of MEF2C on cognitive function. (A) Schematic diagram of the experimental design. Total exploration time (B) and discrimination index (C) in the NOR test. Escape latency (D) and average swimming speed (E) during the acquisition phase. Two‐way ANOVA followed by Bonferroni's multiple comparisons test. **p < 0.01, C versus AS; ^##^ *p* < 0.01, AS versus AS + MEF2C. ^&^ *p* < 0.05, ^&&^ *p* < 0.01, AS versus AS + RSL3; ^$^ *p* < 0.05, ^$$^ *p* < 0.01, AS + MEF2C versus AS + MEF2C + RSL3. (F) The trajectory of mice following the indicated treatments. The red circle denotes the hidden platform, the red and blue dots denote the start and end of swimming, respectively. Time spent in the target quadrant (G) and platform crossing times (H) during the space exploration test of the MWM test. One‐way ANOVA followed by Tukey's post hoc test. *p < 0.05, **p < 0.01. Data were denoted as mean ± SEM (*n* = 12).

The NOR and MWM tests were performed to ascertain whether the pro‐cognitive properties of MEF2C were abrogated by intraperitoneal treatment with the GPX4‐specific inhibitor RSL3. No significant differences in total exploration time were noted among the five groups in the NOR test (Figure 5B). A further diminution of novel object exploration time was observed in POCD mice treated with RSL3. The discrimination index of *Mef2c*‐OE mice treated with RSL3 was lower when exposed to isoflurane and laparotomy than untreated *Mef2c*‐OE mice (Figure 5C). POCD mice receiving intraperitoneal injection of RSL3 spent more time reaching the hidden platform on days 3, 4, and 5 than untreated POCD animals. The escape latency was significantly prolonged in the AS + MEF2C + RSL3 group compared with that in the AS + MEF2C group (Figure 5D). There were no differences in the average swimming speed among the five groups, indicating that anesthesia/surgery, regardless of RSL3 pretreatment, did not alter the locomotor activity of the rodents (Figure 5E). Preemptive RSL3 administration further deteriorated the spatial memory of mice after anesthesia and surgery, as manifested by the shorter cumulative duration in the target quadrant and decreased platform crossing frequency compared with the AS group. *Mef2c*‐OE mice treated with RSL3 showed diminished performance in spatial memory tasks compared with the AS + MEF2C group (Figure 5F–H). These findings underscore that MEF2C confers postoperative cognitive benefits through a GPX4‐dependent mechanism.

### 3.6 Inhibition of GPX4 Reversed the Anti‐Ferroptotic Property of MEF2C

**FIGURE 6:**
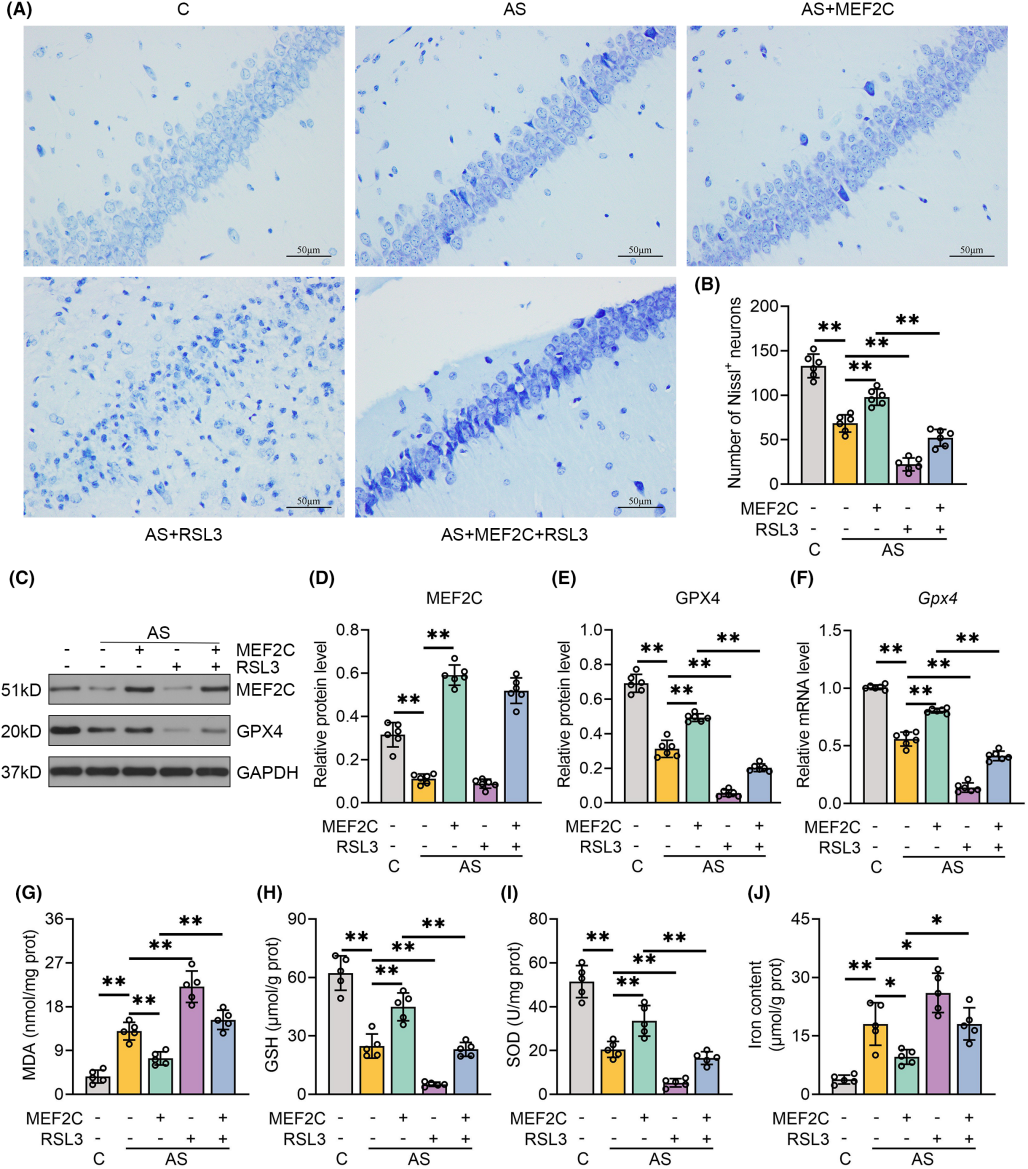
RSL3 neutralized the anti‐ferroptotic property of MEF2C in POCD. (A, B) Representative Nissl staining images in CA1 hippocampus (400×, bar: 50 μm) and the number of Nissl‐positive neurons. (C) Representative gel images of MEF2C and GPX4 in the hippocampus following the indicated treatments. (D, E) Quantification results of MEF2C and GPX4 normalized to GAPDH. (F) RT‐qPCR analysis of GPX4 mRNA expression in the hippocampus of mice following the indicated treatments. (G–J) MDA, GSH, SOD, and iron levels in the hippocampus of mice following the indicated treatments. Data were denoted as mean ± SEM (*n* = 6 or 5). *p < 0.05, **p < 0.01, one‐way ANOVA followed by Tukey's post hoc test.

Nissl staining was performed to evaluate the neuronal survival in the bilateral hippocampal CA1 regions. The vacuole‐like neurons in the control group demonstrated organized and compact arrangements with clear nuclei. The number of Nissl‐positive neurons in the CA1 region significantly decreased following anesthesia and surgery compared with the control group. Nissl^+^ neurons were markedly increased in the hippocampus of the AS + MEF2C group compared with those in the AS group. Conversely, the AS + RSL3 group displayed more pronounced neuronal lesions, including neural loss and irregular, sparse arrangements. Notably, the survival rate of hippocampal neurons reduced after the combined treatment of MEF2C overexpression and RSL3 compared with the AS + MEF2C group (Figure 6A,B). RSL3 intervention did not alter MEF2C but downregulated GPX4 mRNA and protein levels. When compared to the AS + MEF2C group, concomitant regimens of AAV9‐*Mef2c* and RSL3 repressed GPX4 transcription and translation (Figure S4, Figure 6C–F). Preemptive RSL3 administration further augmented MDA and iron accumulation in the hippocampus of mice subjected to isoflurane anesthesia and laparotomy. Further restraint of GSH and SOD levels was observed in the AS + RSL3 group compared with those in the AS group. Moreover, RSL3 antagonized the anti‐ferroptotic effects of MEF2C, as evidenced by elevated levels of MDA and iron, along with decreased levels of GSH and SOD in the AS + MEF2C + RSL3 group compared with the AS + MEF2C group (Figure 6G–J). Taken together, MEF2C protects against ferroptosis in the pathogenesis of POCD in a GPX4‐dependent manner.

## 4 Discussion

Our study linked a hyperactive ferroptosis response with diminished MEF2C abundance in POCD. MEF2C overexpression alleviated anesthesia/surgery‐elicited memory deterioration, reduced lipid peroxidation and ferrous ion levels, and rendered potent antioxidant capacity. Inhibiting GPX4 with RSL3 reversed the anti‐ferroptotic phenotypes mediated by MEF2C. Mechanistically, MEF2C directly bound to the GPX4 promoter and facilitated its transcription.

As a MADS Box TF crucial for neurodevelopment, MEF2C is involved in various neuropsychiatric diseases, including autism spectrum disorder, epilepsy, and schizophrenia [39, 40, 41]. *Mef2c* ^+/−^ mice displayed synaptic abnormalities, reduced dendritic complexity, decreased inhibitory synaptic transmission, increased excitatory synaptic transmission, and impaired long‐term potentiation in the hippocampal CA1 region [42]. MEF2C downregulation in peripheral leukocytes and postmortem brain has been causally implicated in the development of AD [43, 44]. In line with earlier evidence for scant expression of MEF2C following isoflurane anesthesia and tibial fracture [20], we also detected exiguous MEF2C mRNA and protein levels in the hippocampus of mice subjected to isoflurane and laparotomy. Consequently, we illuminated the role of MEF2C in hippocampus‐dependent behavioral tasks. Although postnatal mice lacking *Mef2c* did not exhibit a statistical difference in the discrimination index compared with the littermate control mice [45], our NOR results showed that POCD mice receiving preemptive AAV9‐*Mef2c* administration preferred the novel object. Knockout or knockdown of *Mef2c* impaired spatial learning and memory function [42, 44], whereas AAV9 carrying murine *Mef2c* improved cognitive performance in the MWM tasks following pentobarbital and laparotomy challenges [17]. We also found that MEF2C overexpression rendered protection against cognitive disturbances arising from isoflurane and laparotomy.

MEF2C preferentially binds to adenine (A)/thymine (T)‐rich DNA sequences via its highly conserved MEF2 domain in the N‐terminus [46]. For instance, MEF2C directly bound to A/T‐rich sequences in the promoters of ZO‐1 and occludin, thereby reducing blood–brain barrier permeability in AD microenvironment [47]. It also contributed to axonal branching by interacting with the GTTTTATAAAAAGAA sequence in the *Kif2c* promoter [48]. Our research further highlighted that the TGGTTTGTATA sequence within the *Gpx4* promoter had a strong affinity for MEF2C binding.

Excessive iron leads to neurotoxicity, neuroinflammation, and cognitive decline. Sevoflurane has been shown to trigger ferroptosis through three distinct mechanisms: iron regulatory protein 2 (IRP2)/transferrin receptors 1 (TfR1), GPX4, and Acyl‐CoA synthetase long‐chain family member 4 (ACSL4) [9, 49, 50]. System Xc^−^ and GSH depletion have been implicated in isoflurane‐induced ferroptosis [51]. Consistent with the contributory effects of RSL3 or GPX4 knockdown on ferroptosis in hippocampal neurons [9], we found that RSL3 aggravated lipid peroxidation, iron overload, and memory impairment. Emerging evidence implicates that MEF2C silencing repressed GPX4 protein expression in tumor cells under Erastin‐ or RSL3‐stimulated ferroptotic stress [21, 38]. We demonstrated that MEF2C overexpression suppresses ferroptosis by facilitating GPX4 mRNA and protein levels, thereby uncovering another mechanism of MEF2C distinct from its known antiapoptotic and anti‐neuroinflammatory properties [17, 20].

Our study has certain limitations. The diagnosis of POCD is individual‐based in patients but group‐based in rodent studies. Notably, not all surgical population will develop POCD in clinical settings. Nevertheless, repeated evaluations of learning and memory are commonly avoided in the overwhelming majority of animal studies because of concerns that preoperative behavioral training may confound postoperative cognitive data [52]. Assessing cognitive decline from presurgery to post‐surgery in individual animals may better align with clinical scenarios [53], which represents a promising alternative for the determination of POCD in vivo investigations. At least 5 mg/kg i.p. of RSL3 has been employed to induce ferroptosis in studies related to cognitive disorders [28, 54]. However, its efficacy and suitability necessitate additional validation. We observed the direct impact of the GPX4 inhibitor RSL3 on POCD to specifically investigate whether MEF2C overexpression mitigates POCD by transcriptionally activating *Gpx4*, rather than comparing it with other known ferroptosis inducers. Although our previous studies and other works [1, 17] have extensively shown no therapeutic efficacy of AAV9 empty vector in POCD model, the absence of a control vector may complicate data interpretation. Future research will incorporate positive controls to provide a more comprehensive assessment.

In summary, inhibiting ferroptosis represents a promising molecular approach to preventing POCD. Our findings that MEF2C‐mediated upregulation of GPX4 impairs hippocampal sensitivity to ferroptosis highlight MEF2C as a prospective therapeutic target in response to isoflurane and laparotomy challenges.

## Author Contributions

S.W. and Z.W. completed the biochemical experiments and prepared the draft manuscript. S.W., X.B., X.P., and Q.Z. performed the behavioral tests. W.S. and W.W. performed the bioinformatics analysis. W.S., W.G., and W.W. provided the funding acquisitions. W.W. and Z.X. designed the study, supervised the study, and provided explicit research mentorship. All authors have read and approved the manuscript.

## Conflicts of Interest

The authors declare no conflicts of interest.

## Supporting information


Appendix S1

## Data Availability

The data that support the findings of this study are available from the corresponding author upon reasonable request.
