# Supplementary material for: MEF2C Alleviates Postoperative Cognitive Dysfunction by Repressing Ferroptosis
Source: CNS Neurosci Ther. 2024 Sep 30;30(10):e70066. doi: 10.1111/cns.70066 (PMC11442332; doi:10.1111/cns.70066)

**Supplementary information**

MEF2C alleviates postoperative cognitive dysfunction by repressing ferroptosis


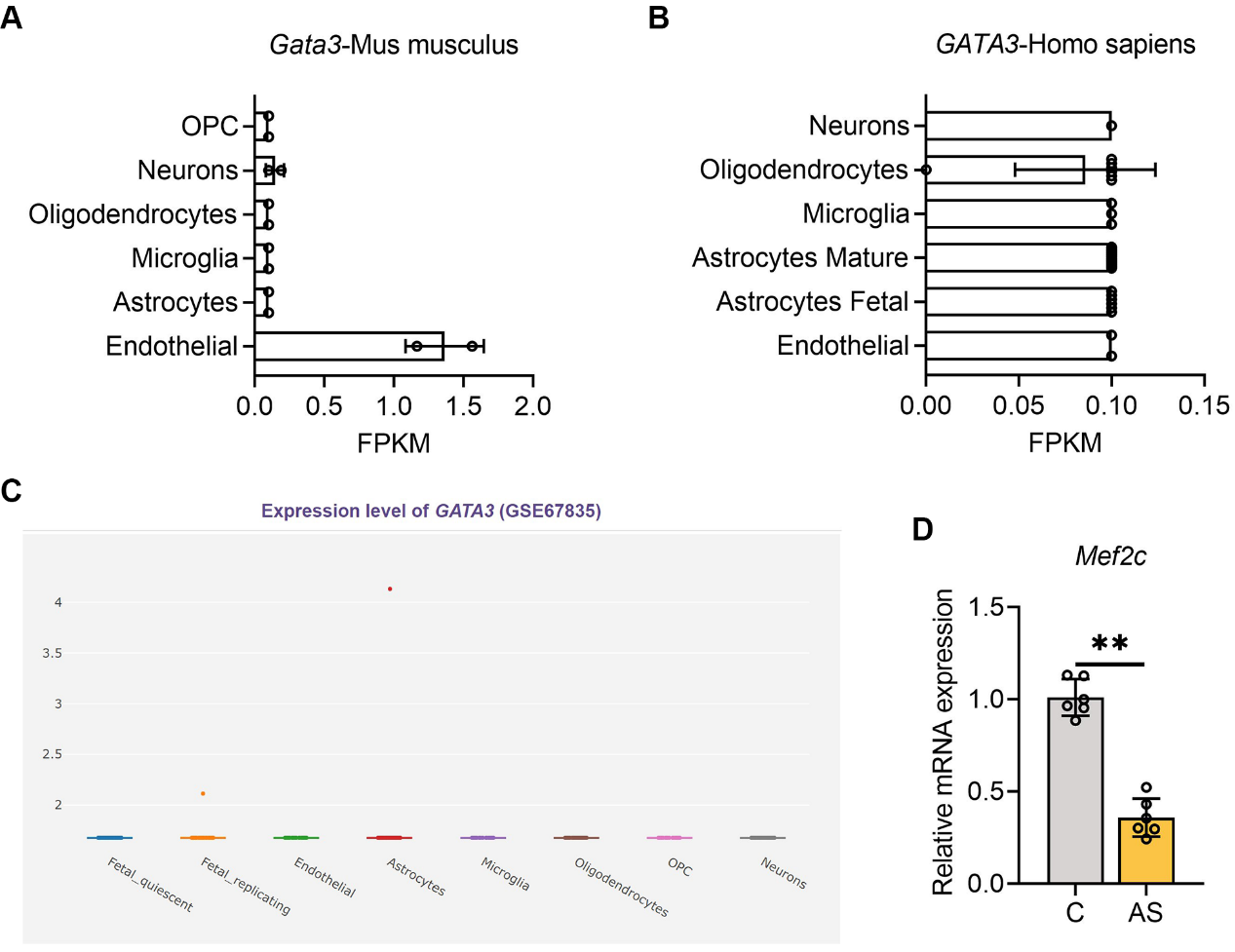


Figure S1. (A-C) According to Brain RNA-Seq (<https://brainrnaseq.org/>) and Alzdata databases (<http://www.alzdata.org/>), single cell RNA-seq of mouse and human brain reveals relative low abundance of *Gata3* in neurons and neuroglia. (D) RT-qPCR analysis of *Mef2c* mRNA expression in the hippocampus of mice following anesthesia and surgery (AS) treatment (n = 6). ^**^*P* < 0.01, unpaired *t*-test.


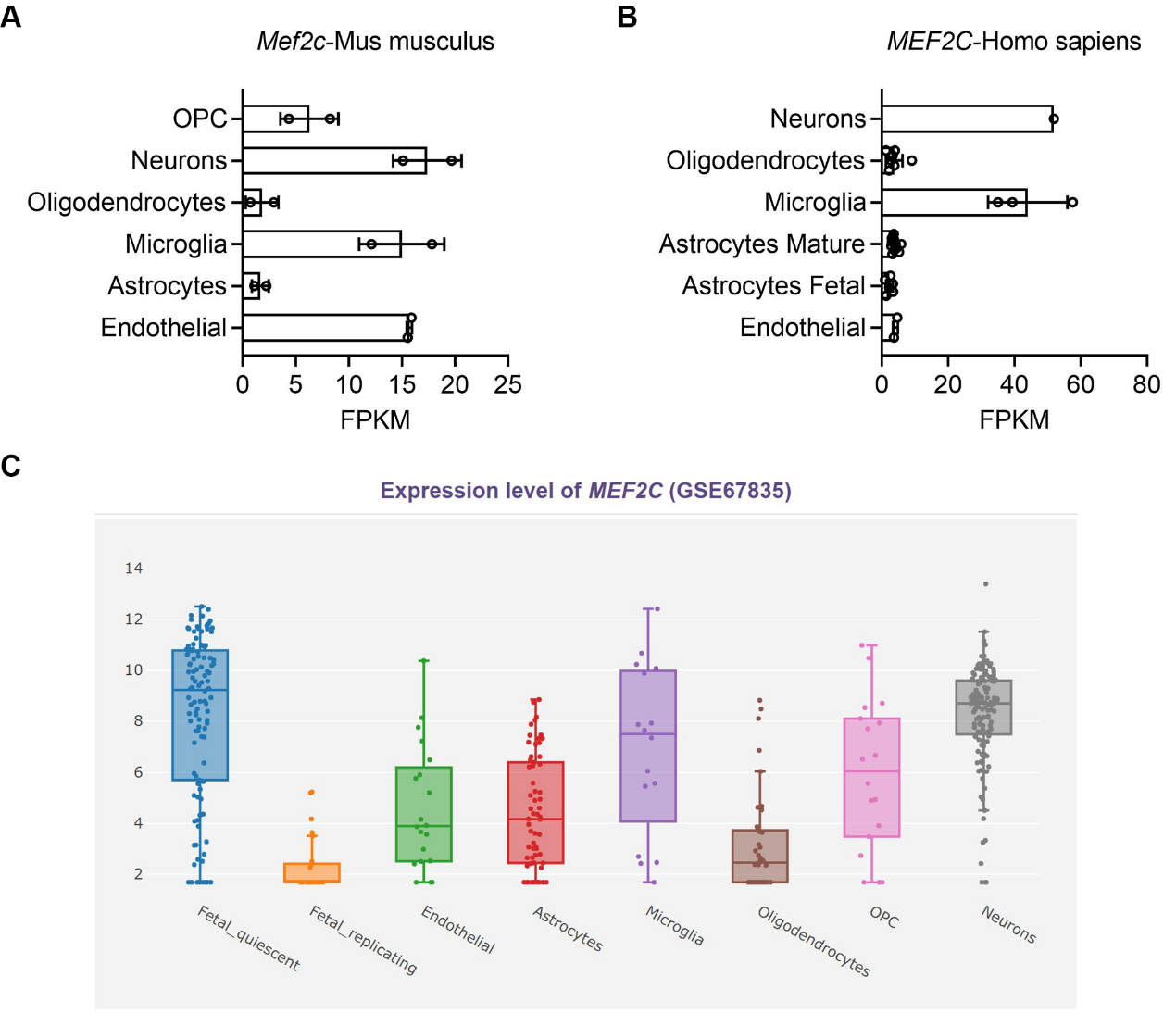


Figure S2. According to Brain RNA-Seq and Alzdata databases, single cell RNA-seq of mouse and human brain reveals that Mef2c is mainly distributed in neurons and microglia.


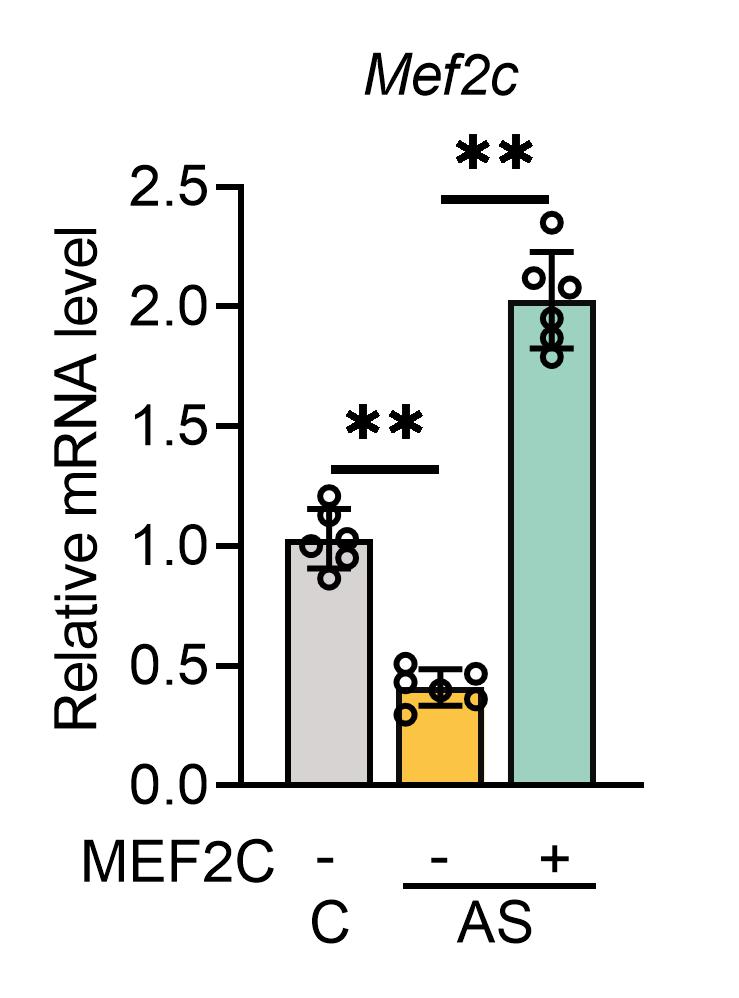


Figure S3. RT-qPCR analysis of *Mef2c* mRNA expression in the hippocampus of mice following the indicated treatments (n = 6). ^**^*P* < 0.01, one-way ANOVA followed by Tukey’s post-hoc test.


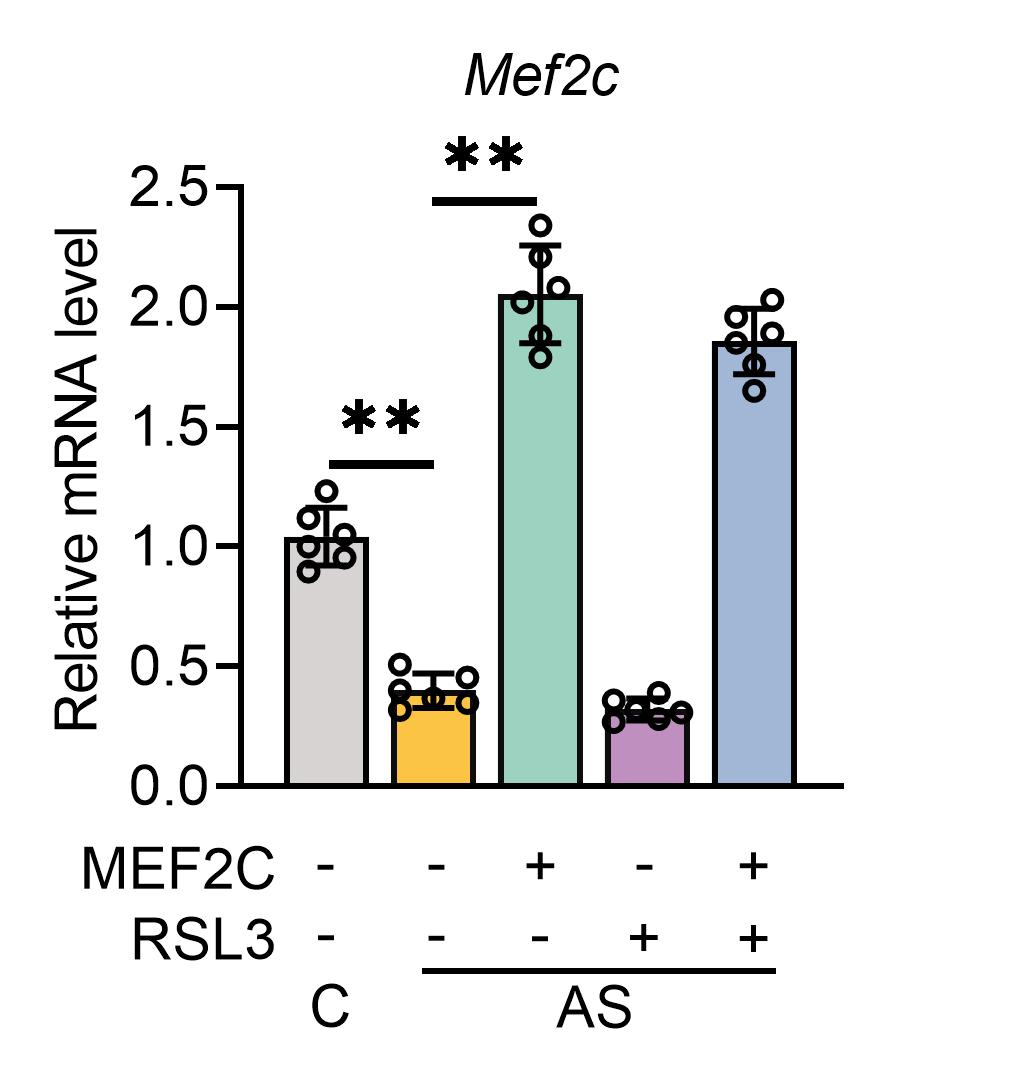


Figure S4. RT-qPCR analysis of *Mef2c* mRNA expression in the hippocampus of mice following the indicated treatments (n = 6). ^**^*P* < 0.01, one-way ANOVA followed by Tukey’s post-hoc test.

**The full uncropped Gels and Blots image(s) as follow:**

Full unedited blot for Figure 1F


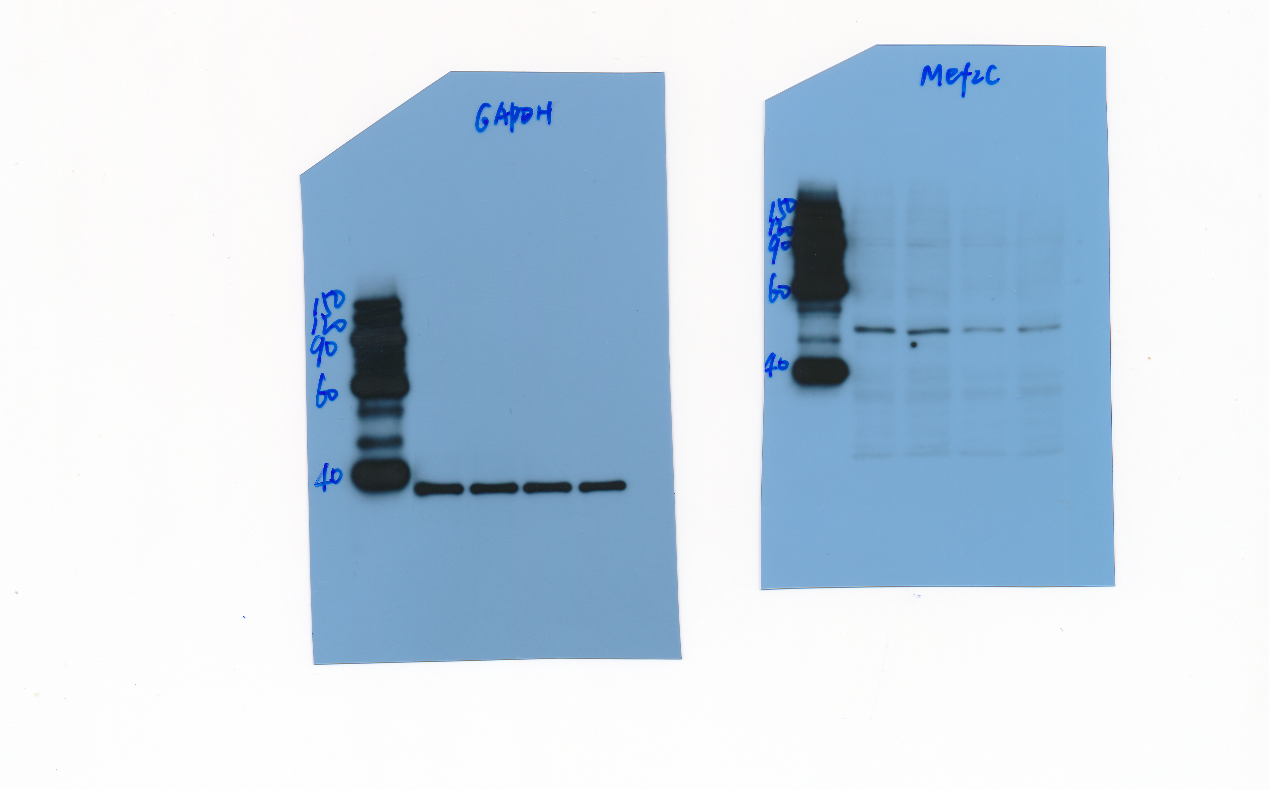


Full unedited blot for Figure 4A


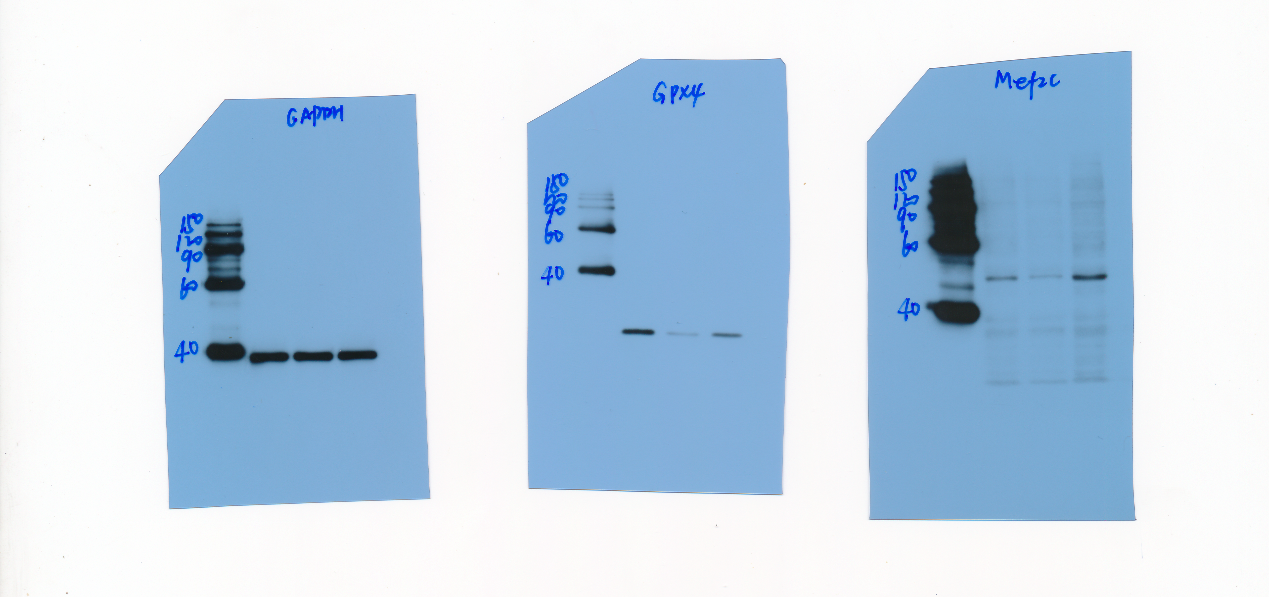


Full unedited blot for Figure 6C


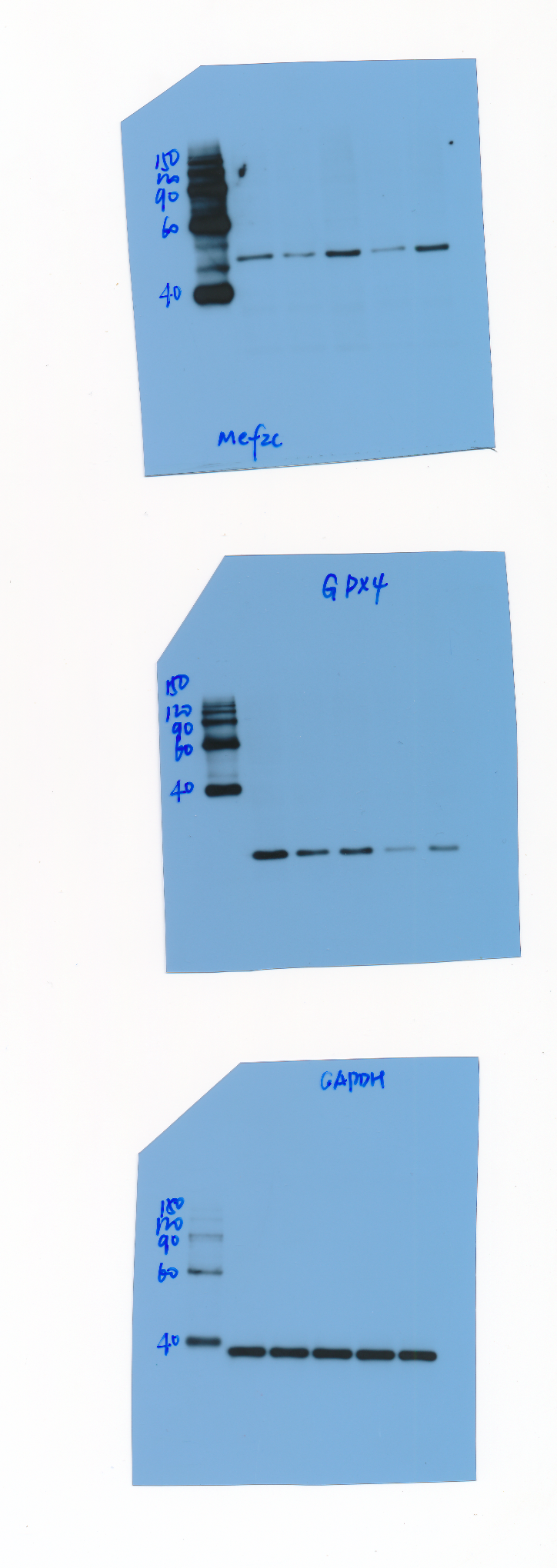

Supplement: Supplementary file 1 — Appendix S1 [file CNS-30-e70066-s001.docx]
